# Molecular Neuropathology of TDP-43 Proteinopathies

**DOI:** 10.3390/ijms10010232

**Published:** 2009-01-09

**Authors:** Manuela Neumann

**Affiliations:** Institute of Neuropathology, University Hospital of Zurich, Schmelzbergstr. 12, 8091 Zurich, Switzerland. E-Mail: Manuela.neumann@usz.ch; Tel. +41 44 255 2849; Fax: +41 44 255 4402

**Keywords:** TDP-43, frontotemporal dementia, amyotrophic lateral sclerosis, molecular neuropathology

## Abstract

The identification of TDP-43 as the major component of the pathologic inclusions in most forms of sporadic and familial frontotemporal lobar degeneration with ubiquitin-positive inclusions (FTLD-U) and amyotrophic lateral sclerosis (ALS) resolved a long-standing enigma concerning the nature of the ubiquitinated disease protein under these conditions. Anti-TDP-43 immunohistochemistry and the recent development of novel tools, such as phosphorylation-specific TDP-43 antibodies, have increased our knowledge about the spectrum of pathological changes associated with FTLD-U and ALS and moreover, facilitated the neuropathological routine diagnosis of these conditions. This review summarizes the recent advances in our understanding on the molecular neuropathology and pathobiology of TDP-43 in FTLD and ALS.

## 1. Introduction

Frontotemporal lobar degeneration (FTLD), the second most common form of presenile dementia, refers to a clinically, genetically and neuropathologically heterogeneous group of neurodegenerative disorders. Clinically, FTLD is characterized by behavioural and/or language dysfunction [1]. In addition some affected individuals manifest with movement disorders such as parkinsonism or motor neuron disease (MND) [2, 3].

While the designation FTLD reflects the prominent frontal and temporal lobe degeneration, the characteristic lesions in the majority of FTLD brains are abnormal ubiquitinated protein inclusions. The protein tau has been identified as the protein building block in the inclusions in about 40% of FTLD cases, and its role in the pathogenesis of neurodegenerative diseases is well established especially after identification of mutation in the microtubule-associated protein tau (*MAPT)* gene in familial forms of FTLD [4]. However, the biochemical composition of the ubiquitinated inclusions in the most common pathological form of FTLD, namely FTLD-U, remained unknown until 2006, when the TAR-DNA binding protein 43 (TDP-43) was identified as the major disease protein in the majority of sporadic and familial FTLD-U cases [5, 6]. Subsequently, the ubiquitinated compact and skein-like inclusions, characteristic for amyotrophic lateral sclerosis (ALS) were also found to be composed of TDP-43 [5, 7], thereby providing strong evidence that both conditions are mechanistically linked and part of a clinico-pathological spectrum of disease, which can be subsumed as TDP-43 proteinopathies [8, 9].

The discovery of TDP-43 not only provided important new insight into the pathogenenic mechanisms underlying FTLD-U and ALS, but also dramatically improved the neuropathological characterization and diagnosis of these conditions. Moreover, it is anticipated that newly developed phosphorylation-specific TDP-43 antibodies [10, 11], allowing the highly sensitive detection of disease-modified TDP-43 species and the specific discrimination between TDP-43 in health and disease, will become the gold-standard in neuropathological routine diagnosis of neurodegenerative diseases. This review highlights the recent advances in our knowledge about the molecular neuropathology and pathobiology of TDP-43 in FTLD and ALS.

## 2. Identification of TDP-43 as disease protein in FTLD-U and ALS

Although extensive efforts have been made for many years in attempts to characterize the biochemical composition of the ubiquitinated inclusions (UBIs) in FTLD-U, they did not prove to be informative. Characterization of UBIs was complicated by the relative low abundance of UBIs, the uneven distribution of UBIs among different FTLD-U cases, and the non-amyloidogenic nature of UBIs as demonstrated by absence of staining with amyloid-binding dyes such as thioflavin S, Congo red, or silver stains. Therefore, an alternative immunologic approach was performed to identify the protein components in the ubiquitinated inclusions in FTLD-U [5, 12]. Briefly, protein extracts enriched for insoluble proteins were generated from postmortem FTLD-U brains and high molecular mass material (Mr>250 kD) was used to immunize mice in order to generate antibodies raised against proteins in UBIs. After screening of thousands of hybridoma supernatants by immunohistochemistry (IHC), novel monoclonal antibodies (mAbs) selectively labeling UBIs were successfully identified. Extensive protein analysis including two-dimensional SDS-PAGE identified protein spots ~25kDa specifically recognized by these mAbs in FTLD-U brains, but not in controls and other neurodegenerative diseases. Subsequently, the resulting peptides obtained by liquid chromatographic mass spectrometry were found to correspond to amino acid sequences in the C-terminal part of a protein known as TDP-43. Commercially available antibodies against TDP-43 consistently labeled the UBIs in sporadic and familial FTLD-U as well as sporadic ALS, but not the characteristic lesions in a variety of other neurodegenerative diseases, thereby confirming and validating TDP-43 as the major protein component of UBIs in FTLD-U and ALS [5]. These findings were quickly confirmed by others [6].

**Figure 1.**
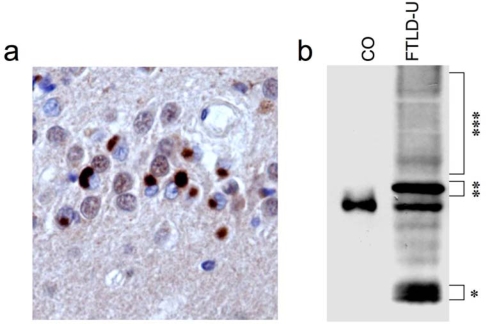
Neuropathology and biochemical alterations of TDP-43 in TDP-43-positive FTLD-U (“FTLD-TDP”). (a) TDP-43 immunohistochemistry labels cytoplasmic inclusions in dentate granule cells in FTLD-U. Note the dramatic loss of normal diffuse nuclear TDP-43 staining in inclusion-bearing cells. (b) Immunoblot analysis of sarcosyl-insoluble protein fractions from TDP-43-positive FTLD-U shows highly characteristic biochemical signature with pathological bands ~25 kDa (*), ~45 kDa (**) and a high molecular smear (***) in addition to the normal TDP-43 ~ 43 kDa.

Most importantly, several disease-associated TDP-43 alterations with potential functional implications have been observed (Figure 1). Thus, TDP-43 inclusion body formation is accompanied by a dramatic change in the subcellular distribution of TDP-43 with complete lack of normal diffuse nuclear TDP-43 staining in inclusion-bearing cells [5]. Biochemical analysis of insoluble protein extracts isolated from affected FTLD-U and ALS tissue revealed a characteristic biochemical profile of TDP-43 with detection of disease-specific bands at ~25kDa, ~45 kDa and a smear of high-molecular-mass proteins in addition to the normal 43 kDa band. Further analysis demonstrated that this profile is due to N-terminal truncation, hyperphosphorylation and ubiquitination of TDP-43 in FTLD-U and ALS [5]. The presence and extent of this pathologic signature in affected brain regions as well as spinal cord roughly corresponds with the density of TDP-43 positive inclusions detected by IHC.

The subsequent identification of 20 mutations to date in the gene *TARDBP* encoding for TDP-43 in ALS (see below) provides further strong evidence for a direct link between TDP-43 dysfunction and neurodegeneration.

## 3. TDP-43 pathology in sporadic and familial forms of FTLD-U

As demonstrated in the initial reports and rapidly confirmed in numerous follow-up studies, TDP-43 is a specific and sensitive marker to detect the characteristic inclusions (neuronal cytoplasmic inclusions (NCI), dystrophic neurites (DN) and neuronal intranuclear inclusions (NII)) in the majority of sporadic and familial FTLD-U cases, including familial FTLD-U forms with mutations in the progranulin (*GRN*) gene, valosin-containing protein (*VCP)* gene and those with linkage to chromosome 9p [5, 13—16]. The absence of binding partners of TDP-43, such as heterogeneous nuclear ribonucleoproteins A1, A2/B1 and C as well as survival motor neuron protein, in these inclusions further emphasizes the specificity of TDP-43 as marker for FTLD-U pathology [17]. Moreover, immunohistochemistry for TDP-43 also allowed the detection of previously unrecognized pathology in FTLD-U. These include sometimes widespread and abundant TDP-43-positive glial cytoplasmic inclusions (GCI) of presumed oligodendroglial lineage [18], more diffuse neuronal cytoplasmic “preinclusions” [19] and dystrophic neurites in the CA1 region in a subset of patients [16]. The majority of these types of lesions are typically ubiquitin-negative, explaining why they have not been recognized previously and furthermore arguing that ubiquitination of TDP-43 might be a late event in inclusion body formation.

The recent development of new antibodies raised against specific TDP-43 epitopes and phosphorylated serine residues have further improved the detection sensitivity of TDP-43 pathology and understanding of inclusion body formation. Thus, immunohistochemical and biochemical analysis using C-terminal and N-terminal specific TDP-43 antibodies have demonstrated that the protein composition in NCIs in cortical brain regions is highly enriched for CTFs, while NCIs in the spinal cord contain more full-length TDP-43, thereby suggesting that inclusion body formation may be regionally different in TDP-43 poteinopathies [20].

Antibodies raised against phosphorylated serine residues (S379, 403, 404, 409 and 410) of TDP-43 further improve and facilitate the neuropathological assessment of TDP-43 pathology, as they only label abnormal TDP-43 in inclusions, but not the often strong physiological nuclear TDP-43 [10, 11].

### 3.1. Heterogeneity among TDP-43-positive FTLD-U cases

**Figure 2.**
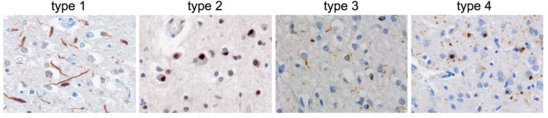
Distinct histological subtypes of TDP-43-positive FTLD-U (“FTLD-TDP”). Immunohistochemistry with antibody against TDP-43 showing the characteristic cortical inclusions in the distinct FTLD-TDP subtypes (numbering according to Sampathu *et al*. [12]).

While UBIs in FTLD-U are generally found in the frontal and temporal cortex as well as in dentate granule cells of the hippocampus in most FTLD-U cases, heterogeneity of FTLD-U pathology with respect to morphology, laminar distribution of ubiquitin and TDP-43 positive inclusions and relative proportion of DNs versus NCIs have been described in two independent studies, leading to the description of four distinct histological FTLD-U subtypes (subtypes 1—4) [12, 15, 21]. Representative images from TDP-43 staining patterns in distinct FTLD-U subtypes are shown in Figure 2 using the classification scheme described by Sampathu *et al* [12]. The characteristic pathological, clinical and genetic features among the four FTLD-U subtypes are summarized in Table 1.

Briefly, subtype 1 histology (also known as subtype 2 in [21]) is characterized by an abundance of long neuritic profiles predominantly in superficial cortical laminae, with few or no NCIs or NIIs. Glial pathology is rare [18].

In subtype 2 cases (also known as subtype 3 in [21]) the predominant inclusions are NCIs in both superficial and deep cortical layers with presence of few neurites, and few or no NIIs. An often more diffuse cytoplasmic staining of “preinclusions” is present. Motor neurons in the hypoglossal nuclei and ventral horn of the spinal cord with TDP-43 positive inclusions similar to that found in pure ALS cases are a common finding, correlating with the fact that patients with subtype 2 histology often represent with additional clinical signs of ALS/MND [21]. Moreover, subtype 2 is often associated with abundant glial pathology in affected cortical, brainstem and spinal cord regions [18]. Neuropathological and biochemical studies on seven cases with genetic linkage to chromosome 9p showed exclusively TDP-43 pathology with morphology and distribution pattern indicative of subtype 2 [15].

The abundance of small neuritic profiles and NCIs, often ring-shaped, predominantly in the superficial cortical layers characterizes subtype 3 histology (also knows as subtype 1 in [21]). Especially in cases with positive family history, moderate numbers of lentiform NIIs can be found in affected cortical regions. Glial pathology is often present in affected cortical regions [18]. While there is variability in the extent of TDP-43 pathology among different brain regions, familial FTLD-U cases with *GRN* mutations exclusively show subtype 3 pathology [15, 22].

The characteristic neuropathological feature of subtype 4 pathology is the abundance of ubiquitin and TDP-43 positive NIIs and DNs with only few NCIs in affected cortical regions and the absence of inclusions in the hippocampal dentate granule cells [14, 23]. So far, all cases with subtype 4 pathology had *VCP* mutations and *vice versa*.

The relevance of the heterogeneity of TDP-43 pathology among FTLD-U cases with respect to pathogenesis remains to be determined. However, the striking correlation of distinct histological subtypes with different genetic forms of familial FTLD-U [15] and its association with distinct clinical syndromes [13, 21] emphasizes the significance of this classification.

### 3.2. Not all FTLD-U cases show TDP-43 pathology

While the initial data suggested that all FTLD-U cases are indeed characterized by TDP-43 pathology, recent follow-up papers on large series of FTLD-U cohorts have shown that there are some important exceptions. Thus, the ubiquitin-positive inclusions in the brains of family members from familial FTLD caused by a mutation in the charged multivesicular body protein (*CHMP2B*) gene do not contain TDP-43 [15, 24]. In addition, a significant number of sporadic FTLD-U cases have now been recognized with absence of TDP-43 pathology [15, 16, 25—27] and a detailed clinico-pathological description of these atypical FTLD-U (aFTLD-U) patients was provided in two recent studies on a total of 15 cases [25, 26]. aFTLD-U patients consistently presented with sporadic early-onset frontotemporal dementia with severe progressive behavioral and personality changes in the absence of aphasia or motor features. Besides TDP-43 negative, ubiquitin-positive NCIs, the most intriguing and most diagnostic pathological finding in aFTLD-U were unique ubiquitin-positive neuronal intranuclear inclusions. Based on the highly consistent clinical and neuropathological phenotype, it was suggested that aFTLD-U represents a new clinico-pathological disease entity.

Since it emerged that the group currently designated as FTLD-U includes at least two distinct entities, a new nomenclature for FTLD was very recently recommended introducing the term FTLD-TDP for TDP-43-positive FTLD-U and FTLD-UPS for those like aFTLD-U, were the ubiquitinated protein(s) remain to be identified [28].

## 4. TDP-43 pathology in sporadic and familial ALS

ALS, the most common form of adult-onset motor neuron disease, is characterized by a loss of upper and lower motor neurons, that results in progressive weakness, muscular wasting, and spasticity. About ~10 % of ALS cases are familial (fALS) and mutations in the Cu/Zn superoxide dismutase (SOD1) gene are thought to be the most common ones accounting for ~20% of fALS [29].

After the initial report that the characteristic lesions obtained in remaining neurons in sporadic ALS (sALS), typically filamentous skeins or compact round inclusions, are composed of TDP-43, the role of TDP-43 in sALS versus fALS was further evaluated. Remarkably, while TDP-43 pathology is a consistent feature in all sALS and non-SOD1-fALS, there is neither histological nor biochemical evidence for TDP-43 pathology in SOD1-fALS cases [7, 30]. Together with the lack of TDP-43 pathology in animal models with SOD1 mutations [31, 32], these data strongly imply that neurodegeneration in SOD1-fALS may results from a different mechanism than that underlying sALS and fALS due to mutations in genes other than SOD1.

In addition to neuronal inclusions, GCIs are a consistent feature in sALS and non-SOD1-fALS [7]. TDP-43 immunohistochemistry also allowed the detection of more extensive and widespread pathology in extramotor regions in patients with ALS with and without dementia [33].

**Figure 3.**
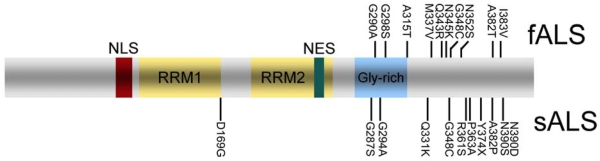
**ALS associated mutations in TDP-43.** Schematic diagram of TDP-43 with characteristic functional domains of TDP-43 and sites of identified mutations in familial (fALS) and sporadic (sALS) amyotrophic lateral sclerosis. Abbreviations: RRM, RNA recognition motif; NLS: nuclear localization sequence; NES, nuclear export sequence.

The presence of TDP-43 pathology in ALS made *TARDBP*, the gene encoding TDP-43 a promising candidate for genetic screening. Initial screens in sporadic FTD and ALS, as well as familial FTD patients failed to identify mutations and no genetic variations could be identified as risk factor for developing FTD or ALS [34–36]. However, subsequent analysis of larger ALS cohorts have now led to the identification of 20 different mutations in 27 unrelated ALS patients (Figure 3), which were absent in healthy controls [37–44]. Twelve mutations were found in fALS following an autosomal dominant trait of inheritance, while others were reported only in sALS cases. Except the Y374X truncation mutation, all other *TARDBP* mutations are missense mutations mostly affecting highly conserved amino residues in the C-terminal region of TDP-43.

Neuropathological data are so far available for the G294A [37], G298S [42], and Q343R [45] *TARDBP* mutation, showing characteristic TDP-43 positive neuronal and glial inclusions in addition to motor neuron loss and presence of Bunina bodies.

Thus far, the functional consequences of *TARDBP* mutations are unknown and need to be investigated in more detail in future studies.

## 5. TDP-43 pathology in other neurodegenerative diseases

Co-occurrence of distinct neurodegenerative disease lesions in the brains of patients with neurodegenerative disorders is an emerging theme in research in these conditions, as exemplified by the high frequency of Lewy body pathology in Alzheimer’s disease (AD) that was only recognized after the routine use of antibodies against α-synuclein. Analogous, TDP-43 immunohistochemistry now also enables the investigation of the co-occurrence of FTLD-U type TDP-43 pathology in the setting of other neurodegenerative disorders and indeed some degree of TDP-43 pathology has now been reported in a variety of other neurodegenerative diseases besides FTLD and ALS.

Thus, TDP-43 pathology is a highly consistent finding in most cases of ALS-Parkinsonism-dementia complex of Guam [46, 47]. Concomitant TDP-43 pathology has been reported in about 20–30 % of patients with AD, in about 70% of patients with hippocampal sclerosis and in a smaller subset of cases with Lewy body diseases, Pick’s disease, corticobasal degeneration, agryrophilic grain disease and Huntington’s disease [48–54]. Unlike in FTLD, TDP-43 pathology in these conditions is mostly restricted to mesial temporal regions. TDP-43 immunoreactivity is often found in separate inclusions, or only partially colocalizes with characteristic lesions found in these diseases, such as neurofibrillary tangles [51, 53]. The clinical significance of additional TDP-43 in the setting of other neurodegenerative diseases is still uncertain and needs to be further examined in detailed clinico-pathological studies.

## 6. Biology and Pathobiology of TDP-43

TDP-43 is a 414 amino acid protein encoded by the *TARDBP* gene on chromosome 1. It was first cloned as a human protein capable of binding to the transactive response DNA of human immunodeficiency virus type 1 [55], and later identified as part of a complex involved in splicing of the cystic fibrosis transmembrane conductance regulator gene [56] and the apolipoprotein A-II gene [57]. TDP-43 is highly conserved, ubiquitously expressed and predominantly localized to the nucleus under normal conditions. It consists of two RNA recognition motifs and a glycine-rich C-terminal region (Figure 3). The exon skipping and splicing inhibitory activity requires the C-terminal region of TDP-43 by interaction with other members of the heterogeneous nuclear ribonucleoprotein (hnRNP) family [58].

In addition to its well characterized role in transcription and splicing regulation, more recent studies suggest that TDP might be involved in other cellular processes such as in microRNA biogenesis, apoptosis, cell division, mRNA stabilization and regulation of neuronal plasticity by acting as neuronal activity response factor [59–61]. Finally, TDP-43 may act as scaffold for nuclear bodies through interaction with survival motor neuron protein [62].

The mechanistic aspects leading to accumulation of pathologic TDP-43 in cytoplasmic, neuritic, and nuclear inclusions and the functional consequences of TDP-43 accumulation are currently not well understood. The dramatic change in subcellular distribution of TDP-43 from the nucleus to the cytoplasm in affected cells in FTLD and ALS suggests that maybe loss of physiological nuclear TDP-43 function in transcription and mRNA processing might play a pathogenic role. Alternatively, generation and sequestration of abnormal TDP-43 species such as C-terminal fragments and hyperphopshorylated TDP-43 enriched in inclusions might induce a toxic gain of function. Recent studies have shown that TDP-43 continuously shuttles between the nucleus and cytoplasm, a process partially regulated by nuclear localization signal (NLS) and nuclear export signal (NES) motifs [63,64]. Restricting TDP-43 from entering the nucleus by changing the NLS motif in cell culture systems is reported to lead to cytoplasmic TDP-43 aggregates, changes in the solubility of TDP-43 and sequestration of endogenous TDP-43, thereby leading to a depletion of nuclear TDP-43 [63]. Thus, perturbation of the normal shuttling of TDP-43 between nucleus and cytoplasm may predispose to both, the formation of cytoplasmic inclusions and loss of nuclear TDP-43.

It is expected that elucidating the functional consequences of ALS-associated *TARDBP* mutations will provide us with important insights also into the disease mechanisms underlying sporadic ALS and FTLD-U. Currently discussed hypotheses are that *TARDBP* mutations might interfere with protein-protein interaction, which might affect its nuclear transport or well-known transcriptional and splicing activities; or that *TARDBP* mutations might increase the aggregation tendency of TDP-43 perhaps due to increased phosphorylation [41–43].

## 7. Conclusions

TDP-43 is the major disease protein in most forms of FTLD-U and ALS, thereby providing strong evidence that both conditions are part of a clinico-pathological spectrum with common underlying pathomechanisms. The use of anti-TDP-43 antibodies have dramatically improved our knowledge about the spectrum of pathological changes underlying FTLD and ALS and it is highly recommended to implement immunohistochemistry for TDP-43 as routine stain in the neuropathological diagnostics of neurodegenerative diseases. Future research approaches, including development of novel cell culture and animal models, have to determine whether loss of function, toxic gain of function or a combination of both mechanisms contributes to cell death in TDP-43 proteinopathies.

## Figures and Tables

**Table 1.** Heterogeneity among TDP-43-positive FTLD-U (“FTLD-TDP”).

	type 1	type 2	type 3	type 4
**Pathology**	predominance of long neurites, NIIs absent - rare	predominance of cytoplasmic inclusions, often preinclusions, NIIs absent - few	small neurites and cytoplasmic inclusions, NIIs absent - abundant	numerous NIIs and small neurites
*laminar distribution*	upper layer > lower	upper = lower layers	upper layer >> lower	upper layer > lower
*Glial inclusions*	absent - rare	moderate - frequent	moderate - frequent	absent
**Clinical symptoms**	SD	FTD often with MND	FTD or PNFA	IBMPFD
**Genetic defect in familial forms**	/	Chrom 9p	GRN	VCP

Based on the pathological parameters of ubiquitin- and TDP-43-positive inclusions four distinct subtypes (numbering according to Sampathu *et al*. [12]) can be delineated. Notably, there is a striking association of histological subtypes with clinical and genetic parameters. Abbreviations: FTD, frontotemporal dementia; SD, semantic dementia; PNFA, progressive non-fluent aphasia; MND, motor neuron disease; IBMPFD, inclusion body myopathy associated with Paget’s disease of the bone and frontotemporal dementia.
